# Supplementary material for: Patterns of Intron Gain and Loss in Fungi
Source: PLoS Biol. 2004 Nov 30;2(12):e422. doi: 10.1371/journal.pbio.0020422 (PMC532390; doi:10.1371/journal.pbio.0020422)
Supplement: Table S1 — Also available at http://genes.mit.edu/NielsenEtAl/. (4.3 MB ZIP). [file pbio.0020422.st001.zip › NielsenEtAl/html/813.html]

AN3095.1.NCU03576.1.MG03219.1.FG10723.1


```
 CLUSTAL W (1.82) Multiple Sequence Alignments - Introns Inserted


Sequence 1: NCU03576.1	370 aa
Sequence 2: FG10723.1	376 aa
Sequence 3: MG03219.1	394 aa
Sequence 4: AN3095.1	374 aa
Alignment Length: 427 aa
Number Identitical Residues: 155 aa
Alignment Score (without introns) 8536


MG03219.1 	---MYSNWTIQQ--QR2LPSTTRG----------------~-~-----YELTRRTPFLGA
NCU03576.1	MSFLFGRARTRTNVSD~LPRQAREHVVKLDQGPQG-----~K~VEELAKVLSQMKQLLQG
FG10723.1 	MSFLFGRARTRT-VAD~LPKQAREHVLKLE-GPQGPSKVV~T0AEELARVLSQMKTILQG
AN3095.1  	MAFFFNRGRSRQ-PSD~VVRSIKDLLLRLREPSTAS---K0V~EDELAKQLSQMKLMVQG
          	 : ::..   :      :    :       . . ..        .. :  *:: . :: .

MG03219.1 	K--~---D~SESNPDQVFHLIEGIINEDLLHPLAANLHKLPFESRKDAQFIFSSVFRFRP
NCU03576.1	T--~--HE1QEASLEHQYQLVTGMIEEDLLYLLAINLYRLPFDSRKDTQVIFSYVFRFRP
FG10723.1 	TPD1ISQE~ADTSPEQILQLVTGLIDEDLLHLLAVNLFRLPFESRKDTQVIFSYVFRFRP
AN3095.1  	T--~--QE1LEASTDQVHALVQAMLHEDLLYELAVALHNLPFEARKDTQTIFSHILRFKP
          	.      :  ::. ::   *: .::.****: **  *..***::***:* *** ::**:*

MG03219.1 	ASASTKSDPVALGWVVNQRPQVLLELCRGYDHKESATAAGSVLREVLKH-EGACKVILYD
NCU03576.1	PNAPARAEPLALAYVVERRPQVLIELCKGYDHKESAQHAGTVLKELIKSSEAATAVILHD
FG10723.1 	ATAAPKSDPLALSYVVCNRPQVLVELCRGYDHKESATPAGSVLRELLKN-EAAAAIILYD
AN3095.1  	PHGNS-PDPPVISYIVHNRPEIIIELCRGYEHSQSAMPCGTILREALKF-DVIAAIILYD
          	. . . .:* .:.::* .**::::***:**:*.:**  .*::*:* :*  :    :**:*

MG03219.1 	DGDQPGSSVNGVQAIDRDRPQSGTGVFWKFFDWIDRGSFEVSADAFTTFR0EILTKQKEV
NCU03576.1	DGDEPGSSARGVGAIDRNRKQTGNGIFWSFFDWIDRGSFEVAADAFTTFR0ELLTKHKDL
FG10723.1 	DGDEPGSSSKGLNAIDRDRPQSGRGVFWRFFDWVDKSSFEVAADAFTTFR0ELLTRHKDL
AN3095.1  	QSKE-GEPAIRLTEVQPNVPQRGTGVFWRFFHWIDRGTFELSADAFTTFR~---------
          	:..: *..   :  :: :  * * *:** **.*:*:.:**::********          

MG03219.1 	VPH~YLRVNFDLFCSKYNTVLVQSSSYVTKRQSIKLLGEILLDRSNYSVMTEYVASGEHL
NCU03576.1	VPH~YLQTNFDLFFSKYNSILIQSTSYVTKRQSIKLLGEILLDRSNYNVMTAYVDRGEHL
FG10723.1 	VPR~YLSANFELFFDKYNNILVQSNSYVTKRQSIKLLGEILLDRSNYSVMTAYVDRGEHL
AN3095.1  	-AR1YLATNFDYFFAQFNTFLVQSESYVTKRQSIKLLGEILLDRANYSVMMRYVESGENL
          	 .: ** .**: *  ::*..*:** *******************:**.**  **  **:*

MG03219.1 	KICMNLLRDDRKMVQYEGFHVFK~VFVANPNKSIPVQRILIMNKDKLLNFLSHFLEDRTD
NCU03576.1	KICMNLLRDDRKMVQYEGFHVFK~VFVANPHKSVPVQKILLMNREKLLHFLSHFLEDRTD
FG10723.1 	KICMNLLRDDRKMVQYEGFHVFK~VFVANPHKSVAVQKILLMNRDKLLTFLSHFLEDRTD
AN3095.1  	KLCMKLLRDDRKMVQYEGFHVFK0VFVANPDKSVAVQRILINNRDRLLRFLPKFLEDRTD
          	*:**:****************** ******.**:.**:**: *:::** **.:*******

MG03219.1 	DEQFIDEREFLIKQIRNMPASPVPPQGS2PAVRPPDAKPNLRYDVRQGERREARGRDESL
NCU03576.1	DEQFIDEREFLIKQIRNMPPTPVVPQR-~-------------------------------
FG10723.1 	DEQFIDEREFLIKQIRNMPAVPVPPQR-~-------------------------------
AN3095.1  	DDQFTDEKSFLVRQIELLPKEPIEPSRS~---------------AREPSRSTANTTTVA-
          	*:** **:.**::**. :*  *: *. :                  . .   :     : 

MG03219.1 	DEHMHLLLSSLDDDNG
NCU03576.1	----------------
FG10723.1 	----------------
AN3095.1  	----------------
          	
```
